# Infection palustre de la femme enceinte à Niamey au Niger

**DOI:** 10.11604/pamj.2020.37.365.20034

**Published:** 2020-12-22

**Authors:** Zara Maman Oumarou, Mahaman Moustapha Lamine, Tahirou Issaka, Kamayé Moumouni, Ibrahim Alkassoum, Daou Maman, Mahamadou Doutchi, Soumana Alido, Ibrahim Maman Laminou

**Affiliations:** 1 Service de Gynécologie-Obstétrique, Maternité Issaka Gazobi, Niamey, République du Niger,; 2 Département de Parasitologie, Université Cheick Anta Diop, Dakar, République du Sénégal,; 3 Unité de Paludologie-Entomologie Médicale, Centre de Recherche Médicale et Sanitaire, Niamey, République du Niger,; 4 Département de Médecine, Faculté de Science de la Santé de l´Université Abdou Moumouni, Niamey, République du Niger,; 5 Département de Santé Publique, Faculté de Science de la Santé de l´Université Abdou Moumouni, Niamey, République du Niger,; 6 Département de Médecine, Université de Zinder, Zinder, République du Niger

**Keywords:** Paludisme, femme enceinte, signes cliniques, facteurs de risque, Niger, Malaria, pregnant women, clinical signs, risk factors, Niger

## Abstract

**Introduction:**

le paludisme chez la femme enceinte est un problème majeur de santé publique en Afrique. Il a des conséquences graves aussi bien sur la mère, le fœtus que le nouveau-né. Il est responsable d´un fort taux de morbi-mortalité maternelle et infantile. L´objectif de l´étude est de déterminer la prévalence de l´infection plasmodiale chez la femme enceinte, décrire ses signes cliniques et ses complications éventuelles, analyser les facteurs associés et proposer des mesures de prévention.

**Méthodes:**

il s´agit d´une étude transversale, conduite du 1^er^ juin au 30 novembre 2017 à la Maternité Issaka Gazobi (MIG) de Niamey. Le diagnostic a été fait par microscopie.

**Résultats:**

deux cents quarante-neuf (249) femmes ont été incluses dans cette étude. La prévalence de l´infection plasmodiale était de 36,5% (IC95%; [30,6; 42,9]). La densité parasitaire moyenne était de 177 P/μl (DS: 121; [40; 800]). Toutes les infections étaient à P. falciparum. Un peu plus de soixante-treize pourcent (73,6%). Seules 26,4% (24/91) ont fait un paludisme non compliqué ; 9,6% (6/91) ont avorté ; 38,4% des nouveau-nés avaient un faible poids à la naissance et 26,51% (66/249) ont développé un paludisme congénital. Le taux de létalité était de 1,1% (1/91). Le traitement préventif intermittent (TPI) protège significativement contre le paludisme gestationnel (p=0,01).

**Conclusion:**

l´infection des femmes enceintes par le P. falciparum est très fréquente au Niger. Ce portage est le plus souvent asymptomatique mais peut évoluer vers un paludisme non compliqué ou même sévère. Les principales conséquences sont l´avortement, le faible poids à la naissance, le retard de croissance intra utero, le paludisme congénital et le décès maternel. Le TPI et l´utilisation de la moustiquaire imprégnée d´insecticide à longue durée d´action (MILDA) permettent de prévenir l´infection.

## Abstract (en)

**Introduction:**

malaria during pregnancy is a major public health problem in Africa. It can have serious consequences for mother, fetus and newborn. It is associated with high maternal and infant mortality rate. The purpose of our study was to determine the prevalence of plasmodium infection in pregnant women, describe their clinical signs and potential complications, analyze associated factors, and propose preventive measures.

**Methods:**

we conducted a cross-sectional study at the Issaka Gazobi Maternity Ward (MIG), Niamey, from 1 June to 30 November 2017. Diagnosis was based on microscopic examination.

**Results:**

two hundred and forty-nine (249) women were included in this study. The prevalence of plasmodium infection was 36.5% (IC95%; [30.6; 42.9]). Mean parasite density was 177 P/μl (SD: 121; [40; 800]). All infections were due to P. falciparum. Seventy-three point six percent (67/91) of infected women were asymptomatic. Only 26.4% (24/91) of them had uncomplicated malaria; 9.6% (6/91) had miscarriage; 38.4% of newborns were low birthweight; 26.51% (66/249) developed congenital malaria. Mortality rate was 1.1% (1/ 91). Intermittent preventive treatment (IPT) significantly protected patients against gestational malaria (p=0.01).

**Conclusion:**

in Niger, P. falciparum infection very commonly affects pregnant women. It is most often asymptomatic but it can lead to uncomplicated or even severe malaria. Main consequences include abortion, low birth weight, intrauterine growth retardation, congenital malaria and maternal death. IPT and the use of long-lasting insecticide-treated mosquito nets (LLINs) can prevent infection.

## Introduction

Selon l´Organisation mondiale de la santé (OMS), 30 millions de femmes enceintes vivent dans des zones d´endémie palustre de l´Afrique. Deux cents mille (200 000) nourrissons et 10 000 femmes meurent de paludisme pendant la grossesse chaque année [1]. La maladie est dans cette zone une menace perpétuelle pour le couple mère enfant sans oublier ses conséquences économiques [2]. Le Niger a une population de 21,5 millions d´habitants dont 50% sont des femmes [3]. Le Programme National de Lutte contre le Paludisme (PNLP) a notifié 968 934 cas de paludisme chez les femmes enceintes en 2018. Le rôle du placenta dans le paludisme gestationnel a été récemment élucidé. En effet, les parasites sont séquestrés par le placenta du fait d´une cytoadhérence des hématies parasitées aux syncytiotrophoblastes. Le ligan intervenant dans cette cytoadhérence est la chondroïtine sulfate. Un gène parasitaire, le var2csa coderait pour une protéine située à la surface du globule rouge, celle-ci interagissant ensuite avec la chondroïtine sulfate A [4].

Les femmes primipares sont plus affectées par le paludisme gestationnel que les multipares par manque d´immunité contre les parasites séquestrés dans le placenta [5-7]. Les multipares acquièrent naturellement une résistance au *Plasmodium falciparum* au cours des grossesses successives à mesure qu'elles acquièrent des anticorps contre les globules rouges parasités qui se lient à la chondroïtine sulfate de A du placenta [6]. Les manifestations cliniques du paludisme chez la femme enceinte sont variables selon l´intensité de la transmission et le niveau de prémunition: (I) En zone de paludisme stable, le paludisme gestationnel est le plus souvent asymptomatique. Toutefois, les plasmodies infectent le placenta et engendrent une anémie maternelle, souvent mortelle, même en absence d´une parasitémie périphérique. Cette anémie peut provoquer un retard de croissance in utéro et un faible poids à la naissance engendrant une mortalité infantile [8], (II) En zone de paludisme instable, la mortalité maternelle est plus importante [9]. Une infection palustre pendant la grossesse est associée à une anémie, un risque accru de paludisme sévère et des complications fœto-maternelles graves telles qu´un avortement spontané ou une prématurité [8]. Le paludisme gestationnel est responsable de 20% de morts nés en Afrique subsaharienne [10].

Il y´a très peu de données publiées au Niger sur le paludisme de la femme enceinte. Pourtant, la question est d´actualité puisque le traitement préventif intermittent du paludisme chez la femme enceinte est un axe privilégié de la lutte antipaludique. C´est dans ce contexte, que nous avons conduit une étude transversale à la MIG, pour calculer la prévalence de l´infection plasmodiale chez la femme enceinte, décrire ses manifestations cliniques et les complications éventuelles, déterminer les facteurs de risque et enfin proposer des mesures de prévention.

## Méthodes

**Type d´étude:** il s´agit d´une étude transversale pour calculer la prévalence du paludisme gestationnel, décrire ses manifestations cliniques et ses complications, déterminer les facteurs de risque, et proposer des mesures de prévention.

**Site et période d´étude:** l´étude a été conduite à la Maternité Issaka Gazobi de Niamey, en collaboration avec le Centre de Recherche Médicale et Sanitaire (CERMES) de Niamey. L´étude a été réalisée durant six mois, du 1^er^ juin au 30 novembre 2017.

**Population d´étude:** la population d´étude était composée de 249 femmes venues accoucher à la MIG. Étaient considérées comme cas de paludisme, toute femme ayant une GE positive et une température axillaire supérieure à 37,5.

**Critères d´inclusion:** étaient incluses dans cette étude, toutes les femmes venues pour accouchement à la MIG et ayant consenti librement de participer à l´étude.

**Critères de non inclusion:** n´étaient pas incluses dans l´étude, toutes femmes n´ayant pas de carnet de santé, ou ayant pris un traitement antipaludique il y´a moins de deux semaines, ou ayant reçu une transfusion sanguine il y´a moins de deux semaines, ou ayant une infection associée.

**Echantillonnage:** nous avons utilisé la méthode d´échantillonnage probabiliste simple. Avec une prévalence estimée du paludisme gestationnel de 15%, un intervalle de confiance à 95% et une précision de 5%, la taille de l´échantillon calculée est de 196 patientes.

**Collecte des échantillons:** les données sociodémographiques et les antécédents ont été collectés à partir des carnets de santé. Les variables de la mère qui étaient collectées sont: l´identifiant, l´âge, la provenance, le nombre de consultation prénatale, la parité, la gestité, le traitement préventif intermittent, l´utilisation d´une moustiquaire imprégnée d´insecticide à longue durée d´action, le taux d´hémoglobine, l´hématocrite, les plaquettes et un examen biologique pour évaluer l´infection plasmodiale. Après l´accouchement, un examen clinique complet du nouveau-né était réalisé. Les données collectées chez le nouveau-né sont le poids, la taille, le sexe, la température, la fréquence cardiaque, l´Apgar, le score de Sylverman, le pouls, la fréquence respiratoire, le réflexe de succion, une goutte épaisse et un frottis mince sont aussi confectionnés.

**Diagnostic du paludisme gestationnel et examens biologique sanguins:** la microscopie qui est la méthode de référence de l´OMS pour le diagnostic du paludisme fut utilisée. La lecture des lames était faite au microscope optique, au grossissement 100X. La densité parasitaire était calculée pour 200 leucocytes. Elle est déterminée par la formule suivante: DP= Nombre de parasite compté X 8000/200. Tous les examens biologiques du sang des femmes enceintes furent réalisés au laboratoire de biologie de la MIG au moyen d´un automate.

**Considération éthique:** cette étude a été autorisée par la faculté de science de la santé de l´université de Niamey. Elle fait partie des sujets de thèse de doctorat de l´exercice 2017-2018. L´anonymat et la confidentialité médicale ont été respectés. Le consentement de toutes les mères fut obtenu avant leur inclusion.

**Analyse et traitement des données:** les données ont été saisies et traitées avec le logiciel Épi info 7.0. Les tests de Chi deux de Pearson et de Fisher ont été utilisés pour la comparaison des pourcentages. Un seuil de significativité de 5% a été retenu.

## Résultats

**Caractéristiques de la population d´étude:** deux cents quarante neufs (249) mères ont été incluses dans l´étude. L´âge moyen des mères était de 27 ans (SD: 6,2; [12; 42]). Un peu plus de quatre-vingt-douze pourcent exactement 92,8% (240/249) des femmes provenaient de la Communauté Urbaine de Niamey (CUN) et 7,2% (9/249) provenaient du milieu rural. Plus de quatre-vingt-seize pourcent, 96,8%, (241/249) avaient accouché à la MIG et 3,2% (8/249) étaient référées. Plus de soixante-sept pourcent (67,5%) des femmes n´avaient aucun niveau d´instruction, 4% avaient un niveau primaire, 9,7% avaient un niveau moyen et 18.9% avaient un niveau supérieur. Trente-trois pourcent (33%) des femmes n´avaient qu´un seul enfant et 18.5% avaient deux enfants. Le nombre moyen d´enfant vivant était de 2 (DS=1; [1; 9]). Trente et un virgule trois pourcent (31.3%) des femmes étaient primipares.

**La prévalence du paludisme chez les femmes enceintes:** la goutte épaisse de 91 femmes était positive soit une prévalence de 36,5% (IC95% [30,6; 42,9]). La densité parasitaire moyenne était de 177 P/µl (DS=121; |40; 800]). La densité parasitaire moyenne des primipares était de 164P/μl. Il n´y a pas de différence statistiquement significative de densité parasitaire selon la parité (p=0,06). Toutes les infections étaient à *Plasmodium falciparum*.

**Les signes cliniques du paludisme chez les femmes enceintes et le traitement:** seules 26,4% (24/91) des femmes infectées par le *plasmodium* ont eu de la fièvre. Elles sont atteintes d´un paludisme non compliqué. Un peu plus de soixante-treize pourcent -73,6% (67/91) des femmes infectées étaient asymptomatiques et apyrétiques. Aucun cas de paludisme sévère n´était observé. Soixante-treize virgule six pourcent (73,6%) (67/91) des femmes ont été traitées avec l´artémether luméfantrine et 26,4% (24/91) avec de l´artémether injectable. L´évolution du traitement a été favorable pour 98,9% (90/91) des femmes enceintes. Une seule femme enceinte était décédée, ce qui représente un taux de létalité de 1,1%.

**Les complications du paludisme chez les femmes enceintes:** 9,6% (6/91) des femmes enceintes à goutte épaisse positive ont avorté. Par contre, 4,4% (11/249) ont avorté dans la population d´étude. Tous les accouchements se sont produits entre la 37^e^ et 41^e^ semaine d´aménorrhée. La moyenne de l´hémoglobine chez les femmes enceintes à goutte épaisse positive était 10,7 g/dl (DS=1,6; IC [8,2; 15]) contre 11 g/dl (DS=6,8; IC [4,8; 91]) chez les femmes enceintes à goutte épaisse négative. Il n´y a pas de différence statistiquement significative d´hémoglobine entre les deux groupes (p=0,2). La tendance est la même chez les femmes à goute épaisse positive et ayant la fièvre. L´hématocrite chez les femmes enceintes à goutte épaisse positive était de 30,5% (DS=4,8 IC [23; 45,2]) contre 31,5% chez les femmes enceintes à goutte épaisse négative (DS=5,4, IC [14,2; 43]). Il n´y a pas de différence statistiquement significative d´hémoglobine entre les deux groupes (p=0,1). La tendance est la même chez les femmes à goute épaisse positive et ayant la fièvre (p=0,4). Vingt-six virgule cinq pourcent (26,51%) (66/249) des nouveau-nés ont fait un paludisme congénital infection avec une densité parasitaire moyenne de 101 P/μl (DS: 47,3; [80; 320]). Plus de trente-un pourcent - 31,3% (78/249) des enfants avaient un faible poids à la naissance dans la population d´étude. Chez les femmes ayant une goutte épaisse positive, 38,4% des nouveau-nés avaient un faible poids à la naissance contre 27,2% chez les femmes ayant une goutte épaisse négative. Il y´a une différence statistiquement significative de poids à la naissance entre les femmes à paludisme gestationnel et les femmes enceintes sans paludisme (p=0,04).

**Les paramètres biologiques:** la moyenne de l´hémoglobine chez toutes les femmes enceintes était 11,2 g/dl (DS=5,8; IC [4,8; 91]). La moyenne de l´hématocrite chez toutes les femmes enceintes était de 30,5% (DS=4,8; IC [23; 45,2]). La moyenne des plaquettes était de 208790 (DS=75617; IC [99000; 439000]). Celle des plaquettes chez les femmes enceintes fébriles et à goutte épaisse positive était de 209863 plaquettes contre 212057 plaquettes chez les femmes fébriles et à goutte épaisse négative. Il n´y a pas de différence de plaquettes entre les deux groupes (p=0,8).

**Facteurs associés au paludisme gestationnel:** 95% (237/249) des femmes venaient en consultation prénatales (CPN). 26% (65/249) ont fait trois CPN contre 5% qui n´ont fait aucune consultation prénatale. 73% (182/249) des femmes ont reçu le traitement préventif intermittent dont 34.3% ont reçu une dose, 34.3% ont reçu deux doses et 30,9% ont reçu trois doses de TPI. En effet 31,3% (78/249) des femmes déclarent dormir sous moustiquaire. Le Tableau 1, ci-dessous montre les risques relatifs entre le paludisme gestationnel et un certain nombre de facteurs comme la CPN, TPI et MILDA. La consultation prénatale ne semble pas avoir un impact sur le paludisme gestationnel (P<0,05). Les femmes qui dorment sous MILDA font moins de paludisme gestationnel. En effet, 71,4% des femmes qui ne dorment pas sous MILDA ont fait un paludisme gestationnel contre 28,6%. La MILDA est un facteur protecteur contre le paludisme pendant la grossesse (p=0,2). Le TPI protège significativement contre le paludisme gestationnel (p=0,01). La protection est proportionnelle au nombre de doses. Les femmes qui ont reçu deux ou trois doses de TPI (22%) font moins de paludisme gestationnel que celles qui n´ont rien reçu (34%).

**Tableau 1:** relation entre paludisme gestationnel et la CPN, TPI et MILDA

Variable	Effectif	Paludisme gestationnel (%)	OR (IC95%)	p value
**Consultation prénatale (CPN)**				
**Oui**	81	89%	0,2 ([0,2; 0,9])	<0,05
**Non**	10	11%		
**Utilisation des MILDA**				
**Oui**	26	28,6%	0,9 ([0,7; 1])	0,2
**Non**	65	71,4%		
**Traitement Préventif Intermittent avec la Sulfadoxine Pyriméthamine**				
**Oui**	59	64,8	0,7 ([0,6; 0,9])	**0.01**
**Non**	32	35,2		
**Dose 0**	31	34%		
**Dose 1**	19	20,9%		
**Dose 2**	20	22%		
**Dose 3**	20	22%		

C'est un tableau croisé qui analyse le risque de paludisme chez la femme enceinte et des facteurs comme les consultations prénatales, l'utilisation des moustiquaires imprégnées d'insecticides et le traitement préventif intermittent

## Discussion

Cette étude transversale, réalisée au niveau de la Maternité Issaka Gazobi de Niamey, du 1^er^ juin au 30 novembre 2017, mesure la prévalence de l´infection palustre à l´accouchement, décrit ses caractéristiques cliniques, analyse les facteurs de risque et enfin propose des mesures de prévention. L´étude a été réalisée sur 249 femmes enceintes présentées à la Maternité Issaka Gazobi de Niamey pour accouchement. La prévalence de l´infection plasmodiale était de 36,5% (IC95%; [30,6; 42,9] avec une densité parasitaire moyenne de 177 P/µl (DS=121; [40; 800]). Il n´y a pas de différence statistiquement significative de densité parasitaire selon la parité (p=0,06). Toutes les infections étaient à *Plasmodium falciparum*. Le portage parasitaire de *plasmodium* est très fréquent en Afrique tropicale particulièrement dans les zones de transmission stable du paludisme. Ce portage s´explique par l´affinité du *plasmodium* pour le placenta où il est séquestré grâce à une adhésine qui est la chondroïtine sulfate A. Ce niveau de séquestration est tributaire du niveau de la prémunition de la femme qui lui-même dépend de l´intensité de la transmission. C´est ainsi que la prévalence du portage de *Plasmodium* chez les femmes enceintes au Burkina était de 30% [11], en République démocratique du Congo, elle était de 21% (42/196), dont 26,5% (18/68) chez les primipares et 18,8% (24/128) chez les multipares [12], au Cameroun, elle était de 13,4% [13], au Ghana elle varie de 42,6% à 57,4% selon le site [14], et au Nigéria, la prévalence varie de 60% [15] à 41,6% selon l´intensité de la transmission du site [16].

Le portage du *plasmodium* est le plus souvent asymptomatique chez la femme enceinte même en zone de transmission intense [17]. En effet, 73,6% (67/91) des femmes infectées étaient asymptomatiques. Seules 26,4% (24/91) des femmes infectées avaient une température supérieure à 37,5°C. Aucun cas de paludisme sévère n´a été observé. Tous les cas étaient non compliqués. Les signes cliniques ne permettent pas de prédire l´infection palustre chez les femmes enceintes particulièrement en zone de forte transmission. Les symptômes communs du paludisme ne sont pas de puissants prédicteurs d'infection [18].

En termes de complications, 9,6%(6/91) des femmes enceintes à goutte épaisse positive ont avorté. Les taux d´hémoglobine, l´hématocrite et les plaquettes ne sont pas affectés par le portage parasitaire. Le portage parasitaire est aussi un facteur de risque du paludisme congénital pour les nouveau-nés. En effet, 26,51% (66/249) des nouveau-nés ont développés un paludisme congénital infection avec une densité parasitaire moyenne de 101 P/μl (DS: 47,3; [80; 320]) et 38,4% des nouveau-nés des mères infectées avaient un faible poids à la naissance. Plus de soixante-treize pourcent - 73,6% (67/91) des femmes ont été traitées avec l´artémether luméfantrine et 26,4% (24/91) avec de l´artémether injectable. L´évolution du traitement a été favorable pour 98,9% (90/91) des femmes enceintes. Des études récentes ont montré que les combinaisons thérapeutiques à base d´artémisinine (ACT) peuvent être utilisées chez la femme enceinte [19]. La dihydroartémisinine pipéraquine particulière prévient et traite correctement le paludisme chez la femme enceinte [6]. Une seule femme était décédée. Le taux de létalité de 1,1%.

Le traitement préventif intermittent protège significativement contre le paludisme gestationnel (p=0,01) et la protection est proportionnelle au nombre de doses de TPI. Au Burkina, une étude des facteurs favorisant le paludisme chez la femme enceinte montre que l´infection palustre était significativement plus élevée chez les femmes enceintes non-scolarisées, habitant les quartiers périphériques[2]. Les femmes qui dorment sous MILDA font aussi moins de paludisme gestationnel. En effet, 71,4% des femmes qui ne dorment pas sous MILDA ont fait un paludisme gestationnel contre 28,6%. La MILDA est un facteur protecteur contre le paludisme pendant la grossesse.

L´OMS recommande trois approches pour prévenir le paludisme gestationnel [1]: (I) faire dormir les femmes enceintes et leurs nouveau-nés sous des moustiquaires imprégnées d´insecticide. Les MILDA éliminent efficacement les moustiques pendant au moins trois ans [20] réduisant ainsi la prévalence des anémies. (II) donner un traitement préventif intermittent à base de sulfadoxine-pyriméthamine qui réduit les épisodes de paludisme chez la mère, les risques d'anémie maternelle, la parasitémie placentaire, le faible poids de naissance et la mortalité néonatale [21]. Au moins trois (3) doses de TPI sont recommandées durant chaque grossesse. Le TPI est non seulement un moyen efficace mais aussi et surtout peu coûteux. (III) Prendre en charge rapidement et efficacement les cas de paludisme chez les femmes enceintes. Une goutte épaisse ou un test de diagnostic rapide doit être systématiquement réalisé devant tout accès fébrile d´une femme enceinte [10].

Des nombreuses études ont montré l´impact du TPI dans la prévention du paludisme placentaire telles celle de Lena H *et al*. au Ghana et de Eric BF *et al*. au Cameroun [22, 13]. Cependant, l´émergence de la résistance à la SP risque de compromettre cette stratégie. Au Niger, la prévalence de la quadruple mutation *(pfdhfrAsn51Ileu/ pfdhfrCys59Arg/ pfdhfrSer108Asn/ pfdhpsAla437gly)* est de 26% [23]. Celle de la quintuple mutation *(pfdhfrAsn51Ileu/pfdhfrCys59Arg/pfdhfrSer108Asn/pfdhpsAla437gly/pfhpsLys540Glu)* associée à un niveau élevé de la résistance à la SP est nulle. La SP garde par conséquent son efficacité et demeure utilisable aussi bien en TPI qu´en chimioprévention du paludisme saisonnier. Une des limites de cette étude est de n´avoir pas associé un aspect socio anthropologique car des études ont montré l´importance de l´information, de l´éducation et de la communication sur l´utilisation des méthodes de prévention du paludisme chez la femme enceinte [24, 25].

## Conclusion

L´infection des femmes enceintes par des souches de *Plasmodium falciparum* est très fréquente au Niger. Ce portage est le plus souvent asymptomatique mais peut évoluer vers un paludisme non compliqué ou sévère. Les principales conséquences de cette infection sont l´avortement, le retard de croissance intra utérin, le faible poids à la naissance, le paludisme congénital et le décès maternel. Le traitement préventif intermittent et la moustiquaire imprégnée d´insecticide à longue durée d´action sont des facteurs protecteurs. Ils doivent être assurés à toute femme enceinte.

### Etat des connaissances sur le sujet

- Le paludisme chez la femme enceinte a des conséquences graves aussi bien sur la mère, le fœtus que le nouveau-né;
- Les parasites sont séquestrés par le placenta du fait d´une cytoadhérence des hématies parasitées aux syncytiotrophoblastes grâce à la chondroïtine sulfate A;
- Les femmes primipares sont plus affectées par le paludisme gestationnel que les multipares par manque d´immunité contre les parasites séquestrés dans le placenta.

### Contribution de notre étude à la connaissance

- Cette étude détermine la prévalence du paludisme chez la femme enceinte au Niger;
- Elle décrit aussi les manifestations cliniques et les conséquences du paludisme gestationnel chez la femme et le nouveau-né;
- Enfin, elle analyse les facteurs de risque du paludisme chez la femme enceinte et propose des mesures de prévention.

## Remerciements

Nous remercions Dr. Jean Testa, Directeur Scientifique du CERMES pour avoir lu et corrigé le manuscrit.
